# Inclusion body myositis associated with Sjögren’s syndrome

**DOI:** 10.1007/s00296-012-2556-4

**Published:** 2012-12-12

**Authors:** Maria Misterska-Skóra, Agata Sebastian, Piotr Dzięgiel, Maciej Sebastian, Piotr Wiland

**Affiliations:** 1 Department of Rheumatology and Internal Medicine, Academic Hospital Wroclaw, Borowska Street 213, Wrocław, Poland; 2 Department of Histology and Embryology, Wroclaw Medical University, Chałubińskiego Street 6a, Wrocław, Poland; 3 Department of Minimally Invasive Surgery and Proctology, Academic Hospital Wroclaw, Borowska Street 213, Wrocław, Poland

**Keywords:** Sjögren’s syndrome, Inclusion body myositis, Therapeutic option, Quality of life

## Abstract

Inclusion body myositis (IBM) belongs to the group of idiopathic inflammatory myopathies. It is a poorly understood disease, which affects skeletal muscles. IBM usually occurs as an isolated condition, but in some cases, it may be associated with another autoimmune disorder, Sjögren’s syndrome. We report a case of a 47-year-old woman with headaches, symptoms of trigeminal neuralgia, progressive weakness in muscles of the upper and lower extremities and symptoms of dry eyes and mouth. On admission, creatine kinase level was increased to 6,956 IU/mL and lactate dehydrogenase (LDH) to 1,011 U/L in the serum. The increase in inflammatory factor (CRP, ESR) levels was not found. The diagnosis of inclusion body myositis associated with Sjögren’s syndrome was established on the basis of clinical picture and diagnostic tests. In this therapy, methotrexate and methylprednisolone were administered. The considerable improved muscle strength in the upper and lower extremities, improved speech and swallowing, disappearance of headache and reduction in CPK and LDH levels were found 8 months after establishing the diagnosis. Treatment with methotrexate and methylprednisolone improved the clinical symptoms and quality of life of this patient and may offer a therapeutic option for some patients with IBM and concomitant Sjögren’s syndrome.

## Introduction

Inclusion body myositis (IBM) is the most commonly acquired myopathy presenting over the age of 50, mostly in men. IBM belongs to the group of idiopathic inflammatory myopathies. It is a poorly understood disease, which affects skeletal muscles. The diagnosis of IBM should be suspected in an adult who has a disease with slow onset which involves distal and proximal muscles, especially foot extensors and deep flexor tendons. Facial muscles can be affected in IBM leading to the involvement of pharyngeal and neck flexor muscles, causing dysphagia and difficulty in holding up the head. In IBM, the autoimmune features coexist with degenerative features which include vacuolization and intrafiber deposition of amyloid and related molecules similar to those seen in Alzheimer's disease [1, 9].

The diagnosis is confirmed by the increase in serum muscle enzyme levels, electromyographic findings and muscle biopsy, with the presence of characteristic structures such as rimmed vacuoles and amyloid deposits. IBM usually occurs as an isolated condition, but in some cases, it may be associated with another autoimmune disorder, Sjögren’s syndrome [2, 3]. Because of the rarity of the disease, lack of controlled trials and resistance to corticosteroids and other immunosuppressive therapies, the treatment for IBM is difficult and provides poor therapeutic benefits in comparison with the expected efficacy.

## Case report

We report a case of a 47-year-old woman, previously operated on for goiter, who was admitted to the Rheumatology and Internal Medicine Department at the Medical University in Wroclaw to diagnose the cause of muscle pain. The anamnesis revealed that for about 1 year, the patient had complained of recurrent headaches in the temporal and occipital region with the feeling of facial numbness, the symptoms of trigeminal neuralgia and progressive hearing disorders. These were the reasons for many neurological consultations and MRI of the head without finding a cause of the reported symptoms. On admittance to the ward, the patient complained of general weakness, lingering headache, increasing pain and weakness in muscles of the upper and lower extremities, inability to climb stairs, disorders of facial expressions, dysphagia and dysarthria, and weight loss due to lack of appetite (12 kg in 6 months). Furthermore, there had been symptoms of dry eyes and mouth for 3 months prior to the admittance.

Pain and weakness of proximal and distal muscles of the upper and lower extremities, heliotrope discoloration of the eyelids, increased tension in calf muscles, symmetrical Raynaud’s phenomenon on the skin of fingers, and features of dry eyes and mouth were found on physical examination. Laboratory tests revealed high levels of creatine kinase (CK 6,956 IU/L, N: 145), lactate dehydrogenase (LDH 1,011 U/L, N: 247), and aminotransferases ALT (197 U/L, N: 0–35) and AST (335 U/L, N: 0–31). The following were also found: antinuclear antibodies (ANA) with a titer of 1: 3,200 in ELISA, high titer of antiTPO antibodies (>1,000 IU/mL, N: 0–5.61), the presence of rheumatoid factor (15.6 IU/mL, N: 0–14), mild hypergammaglobulinemia (1.6 g/dL, N: 0.6–1.2), decreased serum level of complement factor C4 (0.09 g/L, N: 0.1–0.4) and hypercholesterolemia (276 mg/dL, N: 130–200). The increase in inflammatory factor (CRP, ESR) levels was not found.

In MR angiography of the head, only non-specific changes in the subcortical white matter of the frontal lobes were visualized in the form of several small hyperintense foci on T2-weighted scans typical of vascular changes.

Because of the dry eyes and mouth symptoms, the salivary gland of the lip was excised and examined histopathologically. The histopathological picture revealed advanced inflammatory changes. The T score was 4.

The EMG record of the biceps brachii muscle and gastrocnemius muscle revealed the primary muscle damage.

**Fig. 1:**
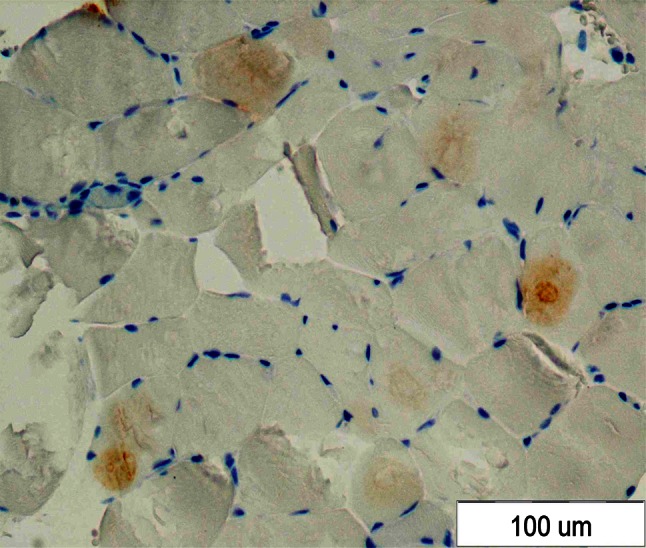
Positive IHC reaction with anti-β amyloid antibodies

**Fig. 2:**
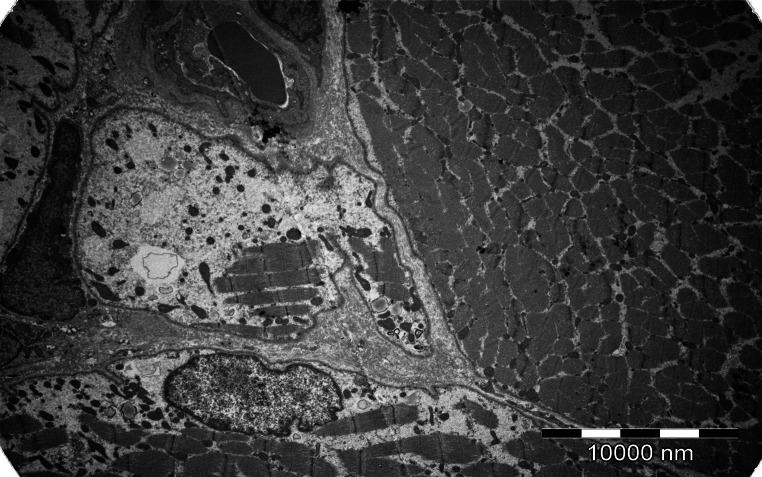
Special features of IBM in electron microscopic examination

The musculocutaneous biopsy from gastrocnemius muscle was taken to establish the diagnosis of myopathy. The histopathological examination was suggestive of inflammatory myopathy which required further assessment using electron microscopy. Electron microscopic and immunohistochemistry examination of a muscle fragment confirmed the diagnosis of IBM (Figs. 1, 2).

The diagnosis of inclusion body myositis associated with Sjögren’s syndrome was established on the basis of clinical picture and diagnostic tests. The patient fulfilled the Griggs et al. criteria for IBM and the EEC consensus criteria for Sjögren’s syndrome. Methotrexate once weekly at a dose of 15 mg and methylprednisolone at a daily dose of 16 mg were administered, which resulted in gradual clinical improvement and reduction in muscle enzyme levels.

The muscle strength improved considerably in the upper and lower extremities; improved speech and swallowing, disappearance of headache and slow reduction in CPK level to 340 IU/L and LDH to 256 U/L were found 8 months after establishing the diagnosis.

## Discussion

Muscle involvement is one of the clinical features of Sjögren’s syndrome. The frequency of muscle changes is estimated at 3 % [3]. IBM, dermatomyositis and polymyositis belong to the forms of immune-mediated myositis [4]. In most cases, the symptoms of dryness precede the symptoms of IBM. Myositis may be associated with extraglandular symptoms such as arthralgias/arthritis, peripheral neuropathy, interstitial kidney disease, primary biliary cirrhosis, cutaneous changes and hepatomegaly. Muscle involvement appears to be symmetrical [2, 4].

The association between IBM and SS has not been found yet. There is a strong association between IBM and the class II major histocompatibility allele HLA-DR3 [5, 6]. There is a major genetic association with allelic markers of the 8.1 MHC ancestral haplotype (HLA-A1, B8, DR3) [6]. In normal muscle fibers, MHC class I or II antigens are not detected. In IBM, MHC expression is the early event that can be detected in this part of muscles with inflammation [7]. It is not clear which aspect, either degenerative or inflammatory, is the primary component in the IBM pathogenesis [8, 9]. Degeneration in muscle leads to the muscle fiber atrophy and fiber apoptosis and causes muscle weakness. In IBM, protein depositions include *β*-amyloid and related molecules similar to those seen in Alzheimer’s disease, suggesting that similar mechanisms lead to tau phosphorylation in both diseases [9]. The proteins can be found inside inclusion or free within the cytoplasma [7]. Currently, there is no one specific molecule that could serve as a unique biomarker for IBM.

PSS induces polyclonal B lymphocyte activation, leading to the production of antibodies that react with antinuclear antibodies (ANA) and soluble nuclear antigens SS-A (Ro) and SS-B (La). The inflammatory infiltrates in salivary glands consist mainly of CD4+ T lymphocytes and, to a considerably smaller extent, CD8+ T and B lymphocytes [10, 11]. The muscle infiltrates in IBM, similar to the Sjögren’s syndrome, consist of a mixture of B and T cells. The proportions of CD8+ and CD4+ cells are various according to the case. Sometimes CD8+  cells are dominant and sometimes CD4+ cells [4]. On the other hand, in both diseases, autoimmune pathogenesis mediated in either case by cytotoxic T cells is present [11]. A common genetic predisposition is linked with MHC in IBM and SS [4].

One of the characteristic symptoms of IBM is dysphagia [12], which Cox et al. found in 65 % of patients with IBM. Dysfunction of the cricopharyngeal sphincter was found in 37 % of patients. Dysphagia during the course of disease is probably caused by the inflammatory involvement of the cricopharyngeal muscles. Facial muscles may be affected in IBM similar to the pharyngeal and neck flexor muscles, causing dysphagia and difficulties holding up the head [7].

Most patients with IBM are unresponsive to treatment with glucocorticoids and conventional immunosuppressive therapy. Current recommendations for the treatment for IBM do not exist. Hydroxychloroquine, azathioprine, methotrexate and methylprednisolone were used, but with poor clinical response and constant, slow deterioration of muscle function [2, 13]. The use of immunoglobulins was connected with improvement in 25 % of patients. This effect was short. IBM has primarily an autoimmune pathogenesis, mediated by cytotoxic T cells [7]. A promising therapy may be the use of alemtuzumab––a T cell-depleting monoclonal antibody. It was used in a small uncontrolled study and showed that the depletion of peripheral T cells also caused the reduction in T cells in the muscle and may stabilize the illness [14]. Physiotherapy is recommended in all the cases.

The evident clinical improvement in the reported case resulted probably from a good response to treatment for concomitant Sjögren’s syndrome.

Nowadays, early and correct diagnosis of IBM appears to be only a treatment challenge because the current therapy is the treatment of choice.
